# Crosstalk Between the Hepatic and Hematopoietic Systems During Embryonic Development

**DOI:** 10.3389/fcell.2020.00612

**Published:** 2020-07-22

**Authors:** Francisca Soares-da-Silva, Márcia Peixoto, Ana Cumano, Perpetua Pinto-do-Ó

**Affiliations:** ^1^Instituto de Investigação e Inovação em Saúde, Universidade do Porto, Porto, Portugal; ^2^Instituto Nacional de Engenharia Biomédica, Universidade do Porto, Porto, Portugal; ^3^Instituto de Ciências Biomédicas Abel Salazar, Universidade do Porto, Porto, Portugal; ^4^Lymphocytes and Immunity Unit, Immunology Department, Pasteur Institute, Paris, France; ^5^INSERM U1223, Paris, France; ^6^Université Paris Diderot, Sorbonne Paris Cité, Paris, France

**Keywords:** hematopoietic stem cells, fetal liver, fetal liver microenvironment, fetal hematopoiesis, hematopoietic stem cell expansion, hematopoietic stem cell niche, self-renewal, cytokine signaling

## Abstract

Hematopoietic stem cells (HSCs) generated during embryonic development are able to maintain hematopoiesis for the lifetime, producing all mature blood lineages. HSC transplantation is a widely used cell therapy intervention in the treatment of hematologic, autoimmune and genetic disorders. Its use, however, is hampered by the inability to expand HSCs *ex vivo*, urging for a better understanding of the mechanisms regulating their physiological expansion. In the adult, HSCs reside in the bone marrow, in specific microenvironments that support stem cell maintenance and differentiation. Conversely, while developing, HSCs are transiently present in the fetal liver, the major hematopoietic site in the embryo, where they expand. Deeper insights on the dynamics of fetal liver composition along development, and on how these different cell types impact hematopoiesis, are needed. Both, the hematopoietic and hepatic fetal systems have been extensively studied, albeit independently. This review aims to explore their concurrent establishment and evaluate to what degree they may cross modulate their respective development. As insights on the molecular networks that govern physiological HSC expansion accumulate, it is foreseeable that strategies to enhance HSC proliferation will be improved.

## Introduction

In the adult organism, hematopoietic stem cells (HSCs) constitute a rare and largely quiescent cell population residing in the bone marrow (BM) (Cheshier et al., 1999). The current dogma states that HSCs self-renew to maintain their pool throughout life and reenter cell cycle in response to stress (Wilson et al., 2008). The balance between self-renewal and differentiation in adult BM has been extensively studied, with the identification of different cellular niches and molecular cues as important elements in HSC maintenance and differentiation – reviewed in Crane et al. (2017) and Pinho and Frenette (2019).

During ontogeny, HSCs undergo a high proliferative stage, expanding in the fetal liver (FL), one of the anatomical locations of embryonic hematopoiesis (Ema and Nakauchi, 2000). Therefore, it has long been assumed that the hepatic microenvironment may drive the proliferation of HSCs while sustaining their primary “stemness” hallmark (functional capacity to reconstitute the hematopoietic compartment of irradiated recipients). So far, however, limited information is available on an HSC supportive environment in the FL and the mechanisms conveying these functional properties remain elusive, hindering effective translation into clinical applications.

A thorough dissection of the architecture and cellular organization of the liver is critical to elucidate the nature of the hematopoietic FL niche and disclose the elements (soluble and/or cell-bound signals, cell-cell contact, cell-matrix interactions, physical properties, etc.) contributing for the regulation of HSCs. This review aims to discuss the role of the FL stroma (encompassing all non-hematopoietic FL cells) and explore the interplay of the two fetal systems – hepatic and hematopoietic – in mouse (or otherwise stated) and how they mutually influence their development.

## The Emergence of the Hematopoietic System During Embryogenesis

**FIGURE 1:**
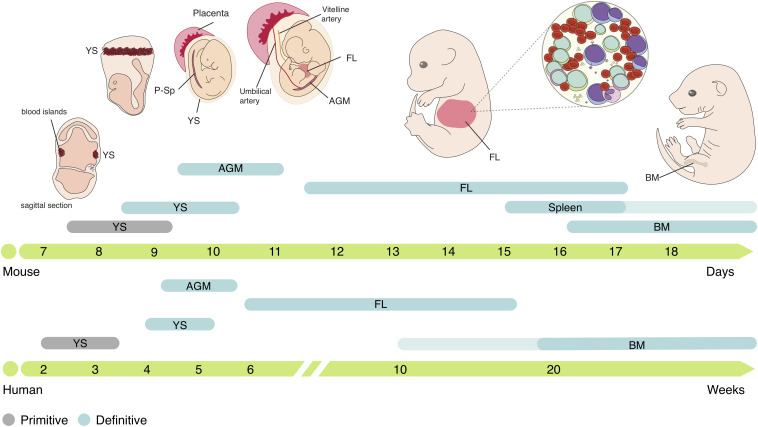
Ontogeny of the hematopoietic system in mouse and human. Embryonic hematopoiesis is established in three distinct waves. The first hematopoietic cells emerge in YS blood islands, generating primitive erythrocytes, macrophages and megakaryocytes, constituting the primitive hematopoietic wave. The second hematopoietic wave initiates in the vascular plexus of the YS, generating EMPs that produce definitive erythrocytes, megakaryocytes, macrophages, neutrophils, granulocytes and mast cells, but lacks HSC activity. EMPs are the origin of tissue-resident macrophages that can persist throughout life. HSCs emerge in the AGM region and migrate to the FL, the major embryonic hematopoietic organ. In FL, HSCs expand and differentiate into all mature blood cell lineages. Small numbers of hematopoietic progenitors also colonize the fetal spleen and are still found few weeks after birth. Migration to the BM, where HSCs reside through adulthood, occurs as a continuous process and, in humans, can take several weeks. YS, yolk sac; P-Sp para-aortic splanchnopleura; FL, fetal liver; AGM, aorta-gonads-mesonephros; BM, bone marrow.

The adult hematopoietic system relies on a robust process whereby HSCs divide and differentiate generating all mature blood lineages. In physiological conditions, this process takes place in the BM in both humans and mice. Even though adult hematopoiesis occurs in the BM, this is merely the end-site of an otherwise thrilling journey through different anatomic locations (Figure 1).

The first hematopoietic cells emerge in the yolk sac (YS) in extra-embryonic structures named blood islands at around embryonic day (E) 7.5 in mice and 2–3 weeks post-conception (wpc) in humans (Bloom and Bartelmez, 1940; Palis et al., 1999). Primitive erythroid progenitors (EryP) generate primitive erythrocytes, large nucleated cells that express embryonic globins (Kingsley et al., 2006), which are found in circulation after the onset of cardiac contractions at E8.25 (∼3 wpc in humans) (Ji et al., 2003; Tavian et al., 1999) and oxygenate the developing embryo. Myeloid progenitors such as macrophage colony-forming cells or megakaryocyte colony-forming cells are also represented during early stages, concomitantly with EryP (Palis et al., 1999), suggesting that primitive hematopoiesis is limited to these three lineages.

Around 24 h later, at E8.5 (4–5 wpc in humans), a second wave of hematopoiesis initiates, with erythro-myeloid progenitors (EMPs) (Migliaccio et al., 1986; Bertrand et al., 2005b) emerging in the vascular plexus of the YS, in a process denominated endothelial to hematopoietic transition (EHT) (Frame et al., 2016; Kasaai et al., 2017). EMPs proliferate and differentiate in the YS into erythroid and myeloid cells but can also be identified in circulation and in the developing liver at later stages (E10.5) via surface expression of c-Kit, CD16/32 and low levels of CD45 (McGrath et al., 2015). These progenitors generate the first definitive erythrocytes, megakaryocytes, macrophages and other myeloid lineages such as neutrophils, granulocytes and mast cells, but lack HSC activity (Palis et al., 1999; McGrath et al., 2015). Although EMPs are a transient population at early stages of embryonic development, they generate different tissue-resident macrophages that, depending on the organ, can persist throughout adulthood (Gomez-Perdiguero et al., 2015), mast cells that are maintained until birth (Gentek et al., 2018), and are the major producers of erythrocytes throughout embryonic life (Soares-da-Silva et al., 2020, Preprint).

A third wave of hematopoiesis occurs between E9.5-E11 in mice (∼4 wpc in humans), with HSCs emergence in the aorta-gonads-mesonephros (AGM) region (Cumano et al., 1996; Medvinsky et al., 1996; Tavian et al., 1996) through EHT (Bertrand et al., 2010; Kissa and Herbomel, 2010). After generation, immature HSCs (imHSCs) undergo a maturation process as they migrate to the FL (Taoudi et al., 2008; Kieusseian et al., 2012) where they proliferate [expanding in numbers by >30-fold (Ema and Nakauchi, 2000)] and differentiate into all blood lineages: erythroid, myeloid and lymphoid. HSCs can be defined by their ability to provide long-term multilineage hematopoietic reconstitution (LTR) when transplanted to lethally irradiated mice (Morrison et al., 1995) and further repopulate secondary recipients. These cells can be found within the Lin^–^CD45^+^Sca1^+^c-Kit^+^ (LSK) compartment and be further divided according to their reconstitution ability in long-term (LSK CD150^+^CD48^–^ LT-HSC) or short-term (LSK CD150^–^CD48^–^ ST-HSC) reconstituting cells (Kim et al., 2006). Downstream progenitors of HSCs such as multipotent progenitors (MPPs), lympho-myeloid-primed progenitors (LMPPs), common lymphoid progenitors (CLPs) and common myeloid progenitors (CMPs) can also be found in FL and are responsible for the seeding of other hematopoietic organs such as the thymus (Ramond et al., 2014). Although adult and embryonic HSCs have similar lineage potentials, some lymphoid lineages are only produced during embryonic development, namely dendritic epidermal T cells (Ikuta et al., 1990), lymphoid tissue–inducer cells (Eberl et al., 2004), and a subset of IL-17-producer γδ T cells (Haas et al., 2012). Embryonic hematopoiesis also takes place in the placenta, starting at E10.5-E11 (∼6 wpc in humans) and declining at around E15.5 (Gekas et al., 2005; Ottersbach and Dzierzak, 2005; Robin et al., 2009). HSCs and other progenitors are also found in the fetal spleen after E15.5 (Christensen et al., 2004), even though without evidence for significant expansion and mostly differentiating into the macrophage lineage (Bertrand et al., 2006). At around E16.5 (∼10 wpc in humans) HSCs migrate to the BM, where they are maintained through adulthood (Charbord et al., 1996; Christensen et al., 2004). In the adult, BM HSCs are largely quiescent (Cheshier et al., 1999) and only divide to maintain the stem cell pool, while the replenishment of blood lineages appears to be guaranteed by downstream MPPs (Sun et al., 2014; Busch et al., 2015; Pei et al., 2017; Rodriguez-Fraticelli et al., 2018).

Thus, embryonic hematopoiesis is characterized by an overlap in time and space of three waves with distinct anatomical origins and lineage potential. All waves converge to the FL, the major hematopoietic organ during embryogenesis.

## The Colonization of the Fl by the Hematopoietic System

### Which Cells Are Present When Hematopoietic Progenitors Arrive?

**FIGURE 2:**
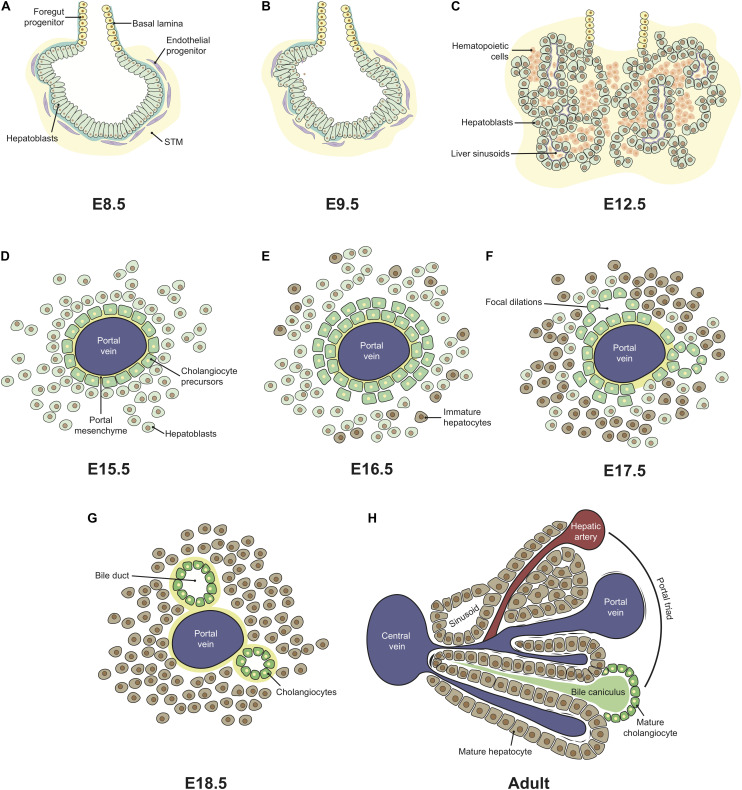
Ontogeny of the fetal liver. Liver development is initiated with the formation of the hepatic diverticulum at E8.5 **(A)**. The single layer of columnar endodermal epithelial cells **(A)** thickens and transitions to a pseudostratified epithelium, followed by the delamination and migration of hepatoblasts into the STM, forming the liver bud by E9.5 **(B)**. Extensive hepatoblast proliferation follows, originating hepatic chords intermingled with the hepatic mesenchyme and surrounding the sinusoids, formed from pre-existing vitelline vessels. From E10.5, hematopoietic cells colonize the FL that rapidly becomes the major fetal hematopoietic organ up until E15.5 **(C)**. From E12.5-E15.5, the FL continues to enlarge, expanding both hematopoietic and hepatic compartments. Structural changes are only evident after hepatoblast-to-cholangiocyte specification occurs around the portal vessels, forming a monolayered ductal plate **(D)** that evolves into a bi-layer at E16.5 **(E)**. Focal dilations **(F)** evolve into bile duct structures in late gestation **(G)**. Portal triads characterize adult liver architecture where a hepatic artery, a portal vein and a bile duct form a complex structure, and a central vein, to which plates of hepatocytes lined by sinusoids converge **(H)**. This architecture is only recognizable few weeks after birth. STM, septum transversum mesenchyme.

As the embryo gastrulates and folds, endoderm envelops the YS ultimately forming a hollow structure, the primitive gut tube, subsequently patterned into foregut, midgut and hindgut regions. The foregut, located in the anterior endoderm, adjacent to the developing heart, generates the liver, alongside with the lungs, thyroid, and pancreas (Tremblay and Zaret, 2005). Embryonic liver development starts at around E8.5-E9 (∼4 wpc in humans) with the formation of the hepatic diverticulum, an extension of the ventral foregut epithelium that invades the septum transverse mesenchyme (STM) and forms a liver bud, by E9.5 (Severn, 1971; Wilson et al., 2006). The liver bud originates from a single-sheet of columnar endodermal epithelium with a gut morphology (Figure 2A), which then transitions to pseudostratified epithelial hepatoblasts (Figure 2B; Bort et al., 2006). At this stage, these cells are separated from the STM by a basement membrane rich in laminin and composed of other extracellular matrix (ECM) molecules, such as nidogen, type IV collagen, fibronectin, and heparan sulfate proteoglycan (Shiojiri and Sugiyama, 2004). A process of extensive hepatoblast proliferation follows, during which the cells outgrow the liver bud, disrupting the basement membrane, into the STM (Figure 2C; Douarin, 1975). The other constituents of the organ, sinusoidal endothelial cells (SECs), mesothelial, sub-mesothelial and hepatic stellate cells have a mesoderm origin (reviewed by Yang et al., 2019), as described below. Angioblasts or endothelial progenitor cells are found delimiting the basement membrane (Figure 2A), resembling a loose “necklace” of cells, and were shown to promote liver organogenesis. In Flk-1^–/–^ mutant embryos, which lack endothelial cells, hepatic specification occurs, but proliferation and migration into the STM are impaired (Matsumoto et al., 2001). At E10.5–E11.0 (∼5–6 wpc in humans), hematopoietic cells colonize the FL that rapidly becomes the major fetal hematopoietic organ (Johnson and Moore, 1975; Migliaccio et al., 1986; Palis et al., 2001).

#### Hepatoblasts

Hepatoblasts are bipotent immature FL cells that differentiate into hepatocytes – the hepatic parenchyma main constituent – or cholangiocytes – the biliary epithelial cells. At the onset of liver development, bipotent hepatoblasts express the liver-specific transcription factors (TFs) hematopoietically-expressed homeobox protein (HHEX) (Bogue et al., 2000), prospero homeobox protein 1 (PROX1) (Dudas et al., 2004), and co-express the hepatocytes’ markers alpha-fetoprotein (AFP), albumin (ALB) (Cascio and Zaret, 1991), cytokeratin 18 (CK18) (Tanimizu et al., 2003), hepatocyte nuclear factor 4α (HNF4α) (Li et al., 2000) and cholangiocytes’ markers such as cytokeratin 19 (CK19) (Tanimizu et al., 2004). Other typical hepatoblast markers are listed in Table 1.

**TABLE 1:** Fetal liver non-hematopoietic compartment: cell types and associated markers.

**Cell type**	**Gene expression**	**Markers used for isolation**
Hepatoblasts	*Hhex, Prox1, Alb, Afp, Ck8, Ck18, Ck19, Met, Hnf6, Oc2, Hnf4a, Ttr, Foxm1, Foxa2, Tbx3, Dlk1, Lgr5, Epcam, Cdh1, Trgb1, Itga6, Liv2, Prom1, Anpep*	DLK1^+^ (Tanimizu et al., 2003; Tan et al., 2017) E-Cadherin^+^ (Nitou et al., 2002; Nierhoff et al., 2005) EpCAM^+^DLK1^+^ (Tanaka et al., 2009) Liv2^+^CD31^–^CD45^–^Lgr5-eGFP^+^ (Prior et al., 2018) Ter119^–^CD45^–^c-Kit^–^CD49f^+^CD29^+^ (Suzuki et al., 2000) VCAM1^+^ALCAM^+^DLK1^+^ (Tsuneto et al., 2013) Ter119^–^CD45^–^CD51^+^VCAM1^+^PDGFRα^–^ (Brouard et al., 2017)

Hepatocytes	*Alb, Afp, Ttr, G6p, Apoa1, Apoh Por, Cps1*	–

Cholangiocytes	*Sox9, Hnf6, Oc2, Spp1, Ck19, Epcam, Krt7*	EpCAM^+^ (Yang et al., 2017)

Endothelial cells	*Flk1, Flt1, Ve-cadh, Pecam1, Mcam, Tek, Tie, Lyve1, Kdr*	CD45^–^Ter119^–^CD31^+^ (Khan et al., 2016) DLK1^–^CD45^–^Ter119^–^CD31^+^LYVE-1^+^ and DLK1^–^CD45^–^Ter119^–^CD31^+^LYVE-1^–^ (Tan et al., 2017)

Mesothelial cells	*Cytokeratin, Cd200, Gpm6a, Alcam, Gp38, Wt1, Podxl, Msln*	Flk1^–^PODXL^*high*^ (Onitsuka et al., 2010)

Sub-mesothelial cells	*Alcam, Desmin, Nestin, p75tnr, Pdgfra, Wt1*	–

Hepatic stellate cells	*Vimentin, Acta2, Desmin, p75NTR, Foxf1, Lhx2, Hlx*	DLK1^–^Ter119^–^CD45^–^CD31^–^LYVE-1^–^p75NTR^+^ (Tan et al., 2017)

Pericytes	*NG2, Nestin, Vimentin, Acta2, Pdgfra, Pdgfrb, Dlk1, Itgav, Endoglin, Vcam, Mcam, Nr2f2*	Ter119^–^CD45^–^CD31^–^Nestin^+^NG2^+^ (Khan et al., 2016) CD45^–^CD56^–^CD34^–^CD146^+1^ (Gerlach et al., 2012)

*^1^Study performed in humans.*

Different markers have been used to isolate hepatoblasts, namely delta like non-canonical Notch ligand [DLK1 or preadipocyte factor 1 (Pref-1)] (Tanimizu et al., 2003), epithelial cadherin (E-cadherin) or CD324 (Nitou et al., 2002), epithelial cell adhesion molecule (EpCAM) or CD326 (Tanaka et al., 2009), and leucine-rich repeat-containing G-protein coupled receptor 5 (LGR5) (Prior et al., 2018). DLK1 is strongly expressed by hepatoblasts in the E10.5 liver bud and continues to be highly expressed until around E16.5, being significantly downregulated thereafter and absent in mature hepatocytes and cholangiocytes (Tanimizu et al., 2003; Tanaka et al., 2009). E-Cadherin, present at the onset of liver outgrowth, is downregulated by the time hepatoblasts migrate to the STM, disrupting the epithelial sheet, although it can be used as a hepatoblast-specific marker after E12.5 (Nitou et al., 2000; Margagliotti et al., 2007). Transient EpCAM expression labels newly formed hepatoblasts but is significantly reduced after E12, while expression after E16 specifically labels bile duct cells (Tanaka et al., 2009). Recently, combining multicolor clonal genetic lineage tracing, organoid cultures and analysis of single-cell RNA sequencing, LGR5 was shown to mark a subpopulation of *bonafide* bipotential hepatoblasts at E9.5–E10 as the origin of the hepatoblast pool (Prior et al., 2018).

#### Endothelial Cells

The main blood vessels in the adult liver are the portal and central veins and the hepatic artery. Up until birth, the hepatic artery is absent and embryonic circulation is sustained by a transient afferent vascular system, the extraembryonic umbilical and vitelline veins (Collardeau-Frachon and Scoazec, 2008). The portal vein arises early in the liver development, between E10.5–E12.5 in mouse (4–6 wpc in human) (Collardeau-Frachon and Scoazec, 2008; Swartley et al., 2016). The hepatic sinusoids are the first vessels to appear, by E10–E10.5, originating from the pre-existing vitelline vessels. The latter sprouts throughout the STM, by angiogenesis, receiving signals from the surrounding mesenchyme (Figure 2C; Swartley et al., 2016). Hepatoblasts were also identified as a positive stimulator of sinusoid morphogenesis and maturation (Takabe et al., 2012). Stabilin 2 (STAB-2) and lymphatic vessel endothelial hyaluronan receptor 1 (LYVE-1) (commonly used as a marker of lymphatics) – hyaluronan receptors – start to be expressed in SECs at E9.5 and E10.5, respectively, and continue to be expressed thereafter (Nonaka et al., 2007; Takabe et al., 2012). Of note, lymphatic vessels were only reported after birth (Swartley et al., 2016). At E9.5, endothelial cells located around the liver diverticulum (Figure 2A) express both CD31/PECAM-1 and Flk-1 (Sugiyama et al., 2010b). CD31 and Flk1 expression in SECs is strong in the early stages of liver development, but is downregulated with time. In adult livers, endothelial cells of portal and hepatic veins strongly express CD31, while it is absent or weakly detected in SECs (Sugiyama et al., 2010b; Takabe et al., 2012). Primitive SECs also strongly express Flk-1, contrarily to endothelial cells of portal and hepatic veins (Sugiyama et al., 2010b). During embryonic liver development, portal vessels express the arterial markers Ephrin-B2 and Neuropilin-1, but not the venous marker EphB4. This expression profile is inverted at the end of gestation, with the transition into a venular phenotype (Wang et al., 1998; Khan et al., 2016). Liver endothelial cells constitute a heterogeneous cellular compartment and different markers should be used for their identification according to vascular location and developmental stage.

#### Mesothelial and Sub-Mesothelial Cells

Mesothelial cells (MCs) compose a single epithelial layer (mesothelium) lining the liver parenchyma on the surface of lobes. From E12.5, MCs are characterized by the expression of cytokeratin, CD200, glycoprotein M6A (GPM6A), podoplanin (PDPN/Gp38), podocalyxin-like protein 1 (PODXL), and mesothelin (MSLN) (Lua and Asahina, 2016). PODXL is highly expressed in immature MCs, being downregulated during development, while MSLN is upregulated. MCs proliferate during liver development and remain quiescent after birth. Wilm’s tumor-1 (WT1) is mainly expressed by MCs (Onitsuka et al., 2010). WT1^–/–^ embryos show incomplete lobulation compared to control littermates at E13.5, reduced numbers of Flk1^–^PODXL^*high*^ MCs, DLK1^+^ hepatoblasts, and total FL cells, suggesting that hepatic development was impaired due to defective MCs (Ijpenberg et al., 2007; Onitsuka et al., 2010). This is supported by the observation that fetal MCs express growth factors (PTN, MDK, and HGF) involved in hepatic development (Onitsuka et al., 2010).

Underneath the MC sheet lays a population of cells expressing Desmin, Nerve growth factor receptor (NGFR/p75NTR) and platelet-derived growth factor receptor α (PDGFRα/CD140a), associated with type IV collagen of the basal lamina, commonly referred as “sub-mesothelial cells” (sub-MC) or capsular fibroblasts. The expression of activated leukocyte cell adhesion molecule (ALCAM/CD166) and WT1 was also observed in MC and sub-MC around E11–E14 and, before that, in the STM by E9–E10 (Asahina et al., 2011; Lua and Asahina, 2016).

#### Hepatic Stellate Cells and/or Pericytes

Although the terms hepatic stellate cells and pericytes have been used by many authors as synonyms, it is not consensual they represent the same population. In adult liver, there is a population of perisinusoidal cells residing in the space of Disse between hepatocytes and SECs, that stores vitamin D lipids (Wake, 1971), and is a major player in liver fibrogenesis (Guyot et al., 2006). MesP1-expressing mesoderm has been considered its earliest ancestry, as it gives rise to the STM – the origin of the liver mesothelium and mesenchymal cells. Migration inward of MC and sub-MC from the liver surface is assumed to give rise to hepatic stellate cells and perivascular mesenchymal cells (Asahina, 2012). Hepatic stellate cells express Desmin, p75NTR, but not the MC markers ALCAM, WT1, and Gp38 (Asahina et al., 2010).

Gerlach et al. (2012) isolated CD146^+^CD45^–^CD56^–^CD34^–^ cells from fetal and adult human livers and identified them as pericytes, a distinct population from hepatic stellate cells. They showed that these cells express NG2 and vimentin, but not GFAP *in situ*, and are found around periportal but not pericentral blood vessels neither within the space of Disse. These cells exhibit high osteogenic and myogenic, but low adipogenic or chondrogenic differentiation potential, in *in vitro* differentiation assays. In mice, a population characterized by the expression of Nestin and NG2 was identified as periportal pericytes, which expresses mesenchymal markers and shows trilineage mesenchymal capacity *in vitro* (Khan et al., 2016).

### Law of Attraction: What Brings Hematopoietic Progenitors to the Developing Liver?

Hematopoietic stem cells emerge from the dorsal aorta directly into circulation and can, therefore, be found in different locations (Cumano et al., 1996; Medvinsky et al., 1996). These cells can travel through the umbilical arteries to the placenta and return to the embryo through the umbilical veins. The umbilical veins drain directly into the liver by fusing with the intrahepatic vascular plexus of the vitelline veins, forming the hepatic sinus. Cells traveling in the right umbilical vein can bypass the liver directly to the vena cava through the *ductus venosus*, a structure only present during fetal development, and can be directed into other embryonic regions through the heart. The liver is also irrigated by the vitelline veins, which transport blood from the YS, and eventually mature to become the portal vein. Another route for newly formed HSCs is to travel from the dorsal aorta through the subcardinal vein to the liver or inferior cardinal vein to the heart. From the heart, circulating cells can reach the developing lungs or upper half of the body (Kiserud, 2005). However, it is in the developing FL that HSCs establish and remain until they migrate to the BM. Transplantation studies show that HSCs can also be found in the placenta (Gekas et al., 2005; Ottersbach and Dzierzak, 2005). The placenta of a mouse model lacking a functional circulatory system was shown to still harbor hematopoietic activity, suggesting that the placenta could generate *de novo* hematopoietic cells with multilineage potential (Rhodes et al., 2008), however, direct evidence for HSC emergence from the placenta is yet to attain.

**FIGURE 3:**
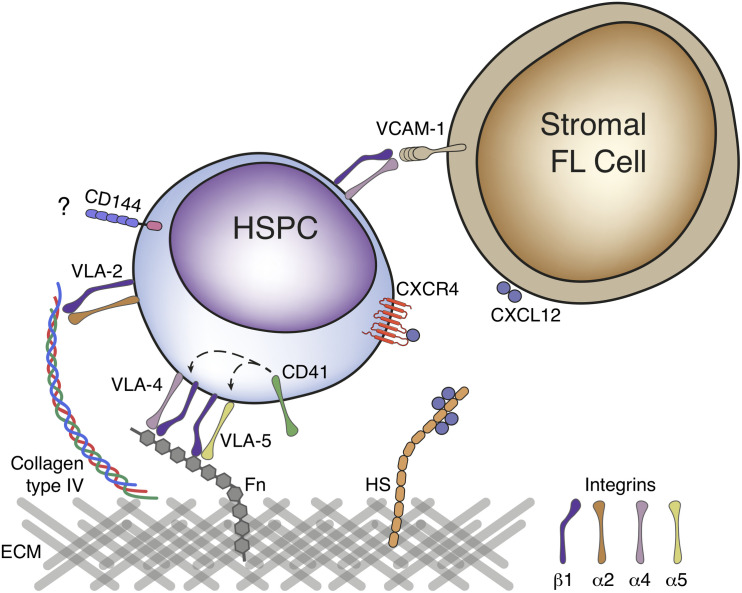
Molecular mechanisms of fetal liver colonization. HSPC express a variety of cell adhesion molecules in their surface that can favor attachment to a specific location. β1 integrin is essential for colonization of FL by HSPC and can bind to the ECM components fibronectin and type IV collagen through dimerization with α4 and α5 or α2 integrins, respectively. VLA-4 and VLA-5 binding to fibronectin has been proposed to be modulated by CD41. VLA-4 (α4β1 integrin) can also mediate cell-cell interactions by binding with VCAM-1, expressed on the surface of FL stromal cells. Expression of the cadherin CD144 in HSCs as they emerge is assumed to play a role in the organ colonization, although no binding partner has been described in FL. CXCL12, a potent chemoattractant is expressed by FL stromal cells and binds to CXCR4, expressed on the surface of HSPC. CXCL12 gradients can be generated as this chemokine binds to proteoglycans in the ECM, namely to heparan sulfate (HS). HSPC, hematopoietic stem and progenitor cell; FL, fetal liver; ECM, extracellular matrix; VLA, very late antigen; Fn, fibronectin; HS, heparan sulfate.

Hematopoietic stem or progenitor cells (HSPCs) but also EMPs, colonize the FL after liver bud formation at E10.5 (Johnson and Moore, 1975; Palis et al., 2001). Distinct mechanisms of FL colonization have been proposed, mostly relying on cell adhesion-mediated processes and/or chemoattraction (cytokines, chemokine signaling, and growth factors) (Figure 3; Hayashi et al., 2019).

Several cell adhesion molecules have been identified in embryonic HSPCs, including integrins, selectins, cadherins, and others. Hematopoietic progenitors express vascular-endothelial cadherin (VE-Cadherin/CD144) as they emerge from the YS (in the case of EMPs) or AGM (in the case of HSCs) that is downregulated thereafter and undetectable in BM HSCs (Fraser et al., 2002; Taoudi et al., 2005). FL CD34^+^ progenitors express higher levels of the integrins β1 (CD29), α2 (CD49b), and α5 (CD49e), similar levels of integrins α4 (CD49d) and α6 (CD49f), E- and P-selectins (CD62E and CD62P, respectively) and CD11b and CD11c molecules, but lower levels of integrin β2 (CD18), CD11a and CD44 than their adult BM equivalent (Roy and Verfaillie, 1999). Seminal studies showed that HSPCs lacking β1 integrin were unable to colonize the FL but were still present in the circulation and capable of generating all blood lineages, suggesting a role for integrin-mediated cell adhesion in FL colonization (Hirsch et al., 1996). Integrin receptors result from the dimerization of α and β subunits and analysis of the expression of the α chain partner of β1 integrin in LSK progenitors revealed integrins α4 and α6 as the most predominant (Sugiyama et al., 2013). FL CD34^+^ progenitors bind the ECM component fibronectin through the integrin receptors α4β1 and α5β1 (also known as VLA-4 and VLA-5) and this binding has been proposed to be modulated by the integrin α2b (GPIIb or CD41), also expressed in these cells (Figure 3; Roy and Verfaillie, 1999; Emambokus and Frampton, 2003). Other ECM components have been tested for adhesion of FL CD34^+^ progenitors such as type I and type IV collagen and laminin, however, only type IV collagen promotes adherence at levels equivalent to that of fibronectin, through α2β1 integrin (VLA-2) (Roy and Verfaillie, 1999). In the FL, hepatoblasts (defined as DLK1^+^ cells) are the major producers of ECM components, namely vitronectin and fibronectin (Sugiyama et al., 2013). Embryos lacking hepatoblasts can still form the liver bud but die between E10.5–E12.5 (Nishina et al., 1999). These FL show decreased expression of vitronectin and fibronectin that may play an important role in FL colonization by HSCs and YS EMPs, although this role has not been specifically assessed (Sugiyama et al., 2013). Integrins can also mediate cell-cell interactions. Cellular bound counterparts of VLA-4 include the vascular cell adhesion molecule-1 (VCAM-1/CD106), expressed by FL hepatoblasts (Sugiyama et al., 2010a).

Cytokine and chemokine signaling can also stand at the basis of FL colonization. The stromal cell-derived factor-1 (SDF-1), commonly known as CXC chemokine ligand 12 (CXCL12), acts through binding to its receptor CXCR4 present in HSPCs and has been extensively studied in the adult BM – reviewed in Yu and Scadden (2016); and Wei and Frenette (2018). CXCL12 expression is stabilized at the cell surface or in the surrounding ECM through proteoglycans binding, allowing the creation of chemokine gradients essential for cell migration (Schumann et al., 2010). In the FL, CXCL12 is expressed by DLK1^+^ hepatoblasts (Chou and Lodish, 2010) and Nestin^+^NG2^+^ pericytes (Khan et al., 2016). The role of CXCL12 in FL colonization was analyzed using CXCL12^–/–^ embryos. At early stages (E12.5–E14.5), the number of HSCs was similar in the FL of mutant animals and controls. By E16.5 FL HSCs were reduced by more than twofold and an abnormally high number was found in circulation. These observations indicate that CXCL12 is an important factor for retaining HSCs in FL, but not for its initial colonization (Ara et al., 2003).

Owing to the particular architecture of fetal circulation, FL is in an anatomically privileged location. Even if HSCs are traveling directly within the embryo through the subcardinal vein, or the umbilical veins after passing in the placenta, the FL is the first intraembryonic organ they encounter. Whether a passive retainment of circulating cells (e.g., through β1-integrin) occurs, or specific signals directly promote chemoattraction of HSPCs to FL is still unclear.

### How Do the Hepatic and Hematopoietic Cell Types Organize During Development?

The structure of the FL changes dramatically during embryonic development. Crawford et al. (2010) extensively characterized the mouse developing hepatobiliary system from E9.5 to E18.5 creating a histology atlas. At initial stages (E10.5–E12.5), the liver is mainly constituted by a vascular plexus and migrating hepatoblasts that later form hepatic chords. At E11.5, the hepatic sinusoids are wide, which may favor the access and establishment of the newly generated hematopoietic progenitors. From E12.5 onward, the organization of the cells in the FL changes as the frequency of hematopoietic cells increases. At E13.5, the most frequent FL population are nucleated erythrocytes that, at early stages, are located throughout the liver parenchyma, in between the hepatic chords, but after E14.5 more mature enucleated erythroid cells are found within the vessels (Ayres-Silva et al., 2011). At this stage, megakaryocytes and erythroblastic islands, which consist of a central macrophage surrounded by erythroid cells, are also distinguishable in the liver parenchyma. These macrophages are responsible for the phagocytosis of the expelled nuclei during erythroid maturation (Bessis et al., 1978). Megakaryocytes are essential to thrombosis and hemostasis and may be determinant in an organ that is mostly constituted by erythroid cells. Moreover, the developing liver seems to provide a unique microenvironment for the expansion of megakaryocyte progenitors (Brouard et al., 2017). From E13.5 to late gestation, no dramatic changes occur in the histology of the FL. Other hematopoietic cells such as B cell progenitors identified by Pax5 expression, can be found interspersed in the tissue by E12.5 and also forming perivascular aggregates by E18.5. Granulocytes are scattered throughout the tissue from E16.5 onward, concentrating/converging around central veins and in the periphery by E17.5, correlating with the presence of mesenchymal cells and suggesting a crosstalk between these distinct cell types (Ayres-Silva et al., 2011). By this time-point, as hematopoietic cells exit the organ and migrate to the BM, hepatoblasts and hepatocytes regain contact (Crawford et al., 2010).

Disclosure of HSCs distribution within the FL has been hindered by the multi-marker assessment required, i.e., Lin^–^c-Kit^+^Sca1^+^CD150^+^CD48^–^, to phenotypically identify these cells. Hematopoietic progenitors (defined by c-Kit expression) are found in close association with DLK1^+^ hepatoblasts at E14.5 (Sugiyama et al., 2013). Nevertheless, c-Kit^+^ cells could mostly represent erythroid progenitors as they are the most frequent population at this stage (Soares-da-Silva et al., 2020, Preprint). Other approaches include the use of transgenic Ly6a-GFP (labeling Sca1^+^ cells) mice, that together with Runx1 localized HSPCs at E11.5 in close contact with endothelial cells (Tamplin et al., 2015). HSCs, profiled as CD150^+^CD48^–^Lin^–^, have been found in close association with Nestin^+^NG2^+^ pericytes surrounding the portal vessels (Khan et al., 2016). Although this characterization more closely identifies a potential HSC, FL studies using mouse models that directly label HSCs are still missing. Generation of a mouse with a single-color reporter driven by endogenous *Hoxb5* (*Hoxb5*–tri-mCherry), which expression in the BM is limited to LT-HSCs and *in situ* imaging evidenced the close proximity of LT-HSCs with VE-Cadherin^+^ cells (Chen et al., 2016). Recently, another HSC-specific reporter line has been described, yet also only analyzed in the adult bone (Christodoulou et al., 2020).

## The Interplay Between the Developing Hepatic-Hematopoietic Tissues

### How Does the FL Environment Modulate Hematopoiesis? A Role in Maturation, Expansion and Differentiation of HSCs?

**FIGURE 4:**
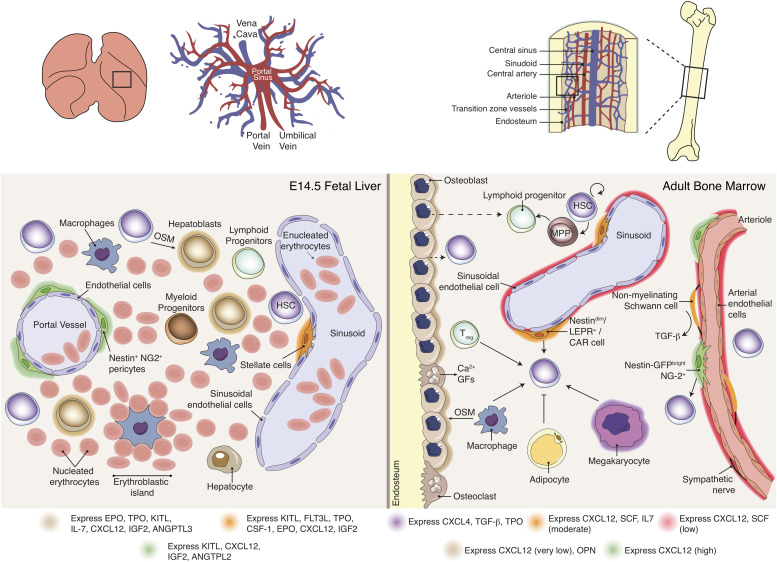
Hematopoietic stem cell niche in fetal liver *versus* bone marrow. Distinct BM stromal cell populations have been described as modulators of HSC functions via expression of soluble and membrane-bound factors. Data available for FL stromal populations is scarce and consequently evidence for a direct role of stromal populations in HSC expansion or differentiation is not reported. The FL microenvironment includes cytokine-producing hepatoblasts, pericytes and stellate cells, together with high number of hematopoietic progenitors and distinct developmental stage erythroid cells. BM Nestin-GFP^*high*^, NG2^+^ perisinusoidal cells and Nestin-GFP^*dim*^, LepR^+^, CAR sinusoidal cells are key regulators of HSC maintenance, contributing differentially for cytokine production. Macrophages, T_*reg*_ and megakaryocytes were also reported to contribute to HSC maintenance and mobilization. Sympathetic nerves regulate HSC mobilization through a tightly control of CXCL12 expression by stromal cells. Non-myelinating Schwann cells activate latent TGF-β into its active form, which may contribute for HSC maintenance. The role of osteoblasts in HSPC is still unclear. Adipocytes may negatively affect HSC maintenance.

Emerging imHSCs lack long-term reconstitution activity in conventional or Rag2^–/–^ immunocompromised mice but can reconstitute NK-deficient Rag2γc^–/–^ animals (Cumano et al., 2001; Bertrand et al., 2005a). After co-culture with the OP9 BM stromal cell line in the presence of thrombopoietin (TPO) or with E10.5 FL rudiments, CD31^+^c-Kit^+^CD45^–^ imHSCs acquire an adult HSC phenotype (LSK CD150^+^CD48^–^) and develop LTR activity in Rag2^–/–^ or conventional mice as they upregulate MHC class I molecules (Kieusseian et al., 2012). These experiments suggest that the FL provides the signals necessary for the maturation of newly formed HSCs. FL stroma also supports the differentiation of committed hematopoietic progenitors towards distinct lineages. Interleukine 7 (IL-7) promotes the survival and proliferation of lymphoid progenitors and controls the determination of the B cell lineage (Sudo et al., 1989; Peschon et al., 1994). In FL, IL-7 is produced by VCAM1^+^ALCAM^+^DLK1^+^ hepatoblasts (Tsuneto et al., 2013) and controls the number of lymphoid progenitors that develop into the B-cell lineage by stabilizing the B-cell transcriptional signature (Berthault et al., 2017). For instance, erythropoietin (EPO) is produced by DLK1^+^ hepatoblasts and is required for proliferation and terminal differentiation of erythroid progenitors (Sugiyama et al., 2011). Also, TPO expressing Ter119^–^CD45^–^CD51^+^VCAM1^+^PDGFRα^–^ FL hepatoblasts support the production of megakaryocytes from adult BM HSCs in a contact-dependent manner (Brouard et al., 2017). TPO is the main regulator of megakaryocyte differentiation and platelet production (Kaushansky, 1995; Eaton and de Sauvage, 1997) but has also been shown to promote survival and proliferation of BM HSPCs *in vitro* (Borge et al., 1996; Ku et al., 1996), the proliferation of fetal hematopoietic progenitors *in vivo* (Alexander et al., 1996) or expansion of BM HSCs following transplantation (Fox et al., 2002). Lack of TPO signaling causes decreased HSC function and numbers (Kimura et al., 1998; Solar et al., 1998), a consequence from the exit of a quiescent state (Nakamura et al., 2007; Qian et al., 2007), possibly leading to a premature exhaustion of the stem cell pool. The survival and proliferation effects of TPO are enhanced when used in combination with other early cytokines, namely FMS-like tyrosine kinase 3 ligand (FLT3L) and c-Kit ligand [KITL, also known as stem cell factor (SCF) or steel factor (SF)] both in murine and human adult BM cells (Ramsfjell et al., 1996; Borge et al., 1997). The highest levels of TPO in the adult are found in the liver (Lok et al., 1994). Systemic TPO produced by hepatocytes, but not by hematopoietic, osteoblast or BM mesenchymal stromal cells is required for BM HSC maintenance (Decker et al., 2018). It can be detected in FL as early as E10.5, having a strong impact on HSC expansion and survival in this organ (Petit-Cocault et al., 2007). Indeed, several cytokines/chemokines/growth factors are important for HSC proliferation, maintenance and survival, namely KITL, FLT3L, insulin growth factor (IGF), angiopoietin-3, angiopoietin-like 2, Wnt family growth factors, Ephrin2a, CSF1, EPO, CXCL12, and IL-6 – reviewed in Sauvageau et al. (2004). In FL, some of these cytokines are expressed by hepatoblasts or other stromal cells, such as stellate cells or pericytes (see Table 2 and Figure 4; Charbord and Moore, 2005; Chou and Lodish, 2010; Khan et al., 2016; Tan et al., 2017).

**TABLE 2:** Cytokine signaling in fetal liver.

**FL Supportive Stroma**		**Hematopoietic progenitors**	
**Pathway involved**	**Expressing cell**	**Receptor**	**Effect**
KITL	DLK1^+^ hepatoblasts (Chou and Lodish, 2010; Sugiyama et al., 2011) Nestin^+^ cells (Khan et al., 2016) Stellate cells (Tan et al., 2017)	c-Kit (CD117) (Yarden et al., 1987)	BM HSC survival and self-renewal (Barker, 1994; Miller et al., 1997)

ANGPTL2	Nestin^+^ cells (Khan et al., 2016)	PirB (Zheng et al., 2012)	BM HSC proliferation (Zhang et al., 2006)

ANGPTL3	DLK1^+^ hepatoblasts (Chou and Lodish, 2010)	PirB (Zheng et al., 2012)	BM HSC maintenance (Zheng et al., 2011) BM HSC proliferation (Zhang et al., 2006)

FLT3L	Stellate cells (Tan et al., 2017)	Flt3 (CD135) (Matthews et al., 1991)	FL HSPC proliferation (Lyman et al., 1993)

TPO	DLK1^+^ hepatoblasts (Chou and Lodish, 2010; Sugiyama et al., 2011; Brouard et al., 2017) Stellate cells (Tan et al., 2017)	MPL (TPO-R, CD110) (Vigon et al., 1992)	FL HSC survival and proliferation (Petit-Cocault et al., 2007) BM HSC quiescence (Nakamura et al., 2007; Qian et al., 2007)

CSF1	Stellate cells (Tan et al., 2017)	Csf-1 Receptor (Guilbert and Stanley, 1980)	Commitment to macrophage lineage (Rieger et al., 2009)

EPO	DLK1^+^ hepatoblasts (Sugiyama et al., 2011) Stellate cells (Tan et al., 2017)	EPO receptor (Sawyer et al., 1987)	Proliferation and differentiation of FL erythroid progenitors (Lin et al., 1996)

CXCL12	Dlk1^+^ hepatoblasts (Chou and Lodish, 2010) Nestin^+^ cells (Khan et al., 2016) Stellate cells (Kubota et al., 2007)	CXCR4 (Bleul et al., 1996)	FL and FBM B-cell lymphopoiesis and FBM myelopoiesis (Nagasawa et al., 1996) BM HSC engraftment post-transplantation (Lai et al., 2014; McDermott et al., 2015) FL HSC retainment (Ara et al., 2003) BM HSC retainment (Sugiyama et al., 2006)

IL-7	VCAM1^+^ALCAM^+^DLK1^+^ hepatoblasts (Tsuneto et al., 2013)	IL-7Rα (Park et al., 1990)	Lymphocyte expansion (Peschon et al., 1994)

IGF2	Dlk1^+^ hepatoblasts (Chou and Lodish, 2010) Nestin^+^ cells (Khan et al., 2016) Stellate cells (Tan et al., 2017)	IGF1-R (Rubin et al., 1983) IGF2-R (Morgan et al., 1987) Insulin receptor (House and Weidemann, 1970)	F L and BM HSC proliferation (Zhang and Lodish, 2004)

The hypothesis that stem cells are regulated by their environment has been proposed by Schofield (1978) and postulates that stem cell properties are maintained by the surrounding cells designated “niche.” Stromal regulation of hematopoiesis has been proposed by many and early studies of co-culture of hematopoietic progenitors with either FL fibroblast or epithelial-stromal cell lines showed erythroid and myeloid support (Tsai et al., 1986; Hata et al., 1993). More than 200 FL stromal cell lines have been developed and tested for maintenance or expansion of HSCs, however, only a few were able to maintain their repopulating activity over a 3-week co-culture period (Wineman et al., 1996). The functional heterogeneity observed was not due to distinct cytokine production as all cell lines expressed similar cytokine profiles, including *Flt3l*, *Kitl*, *Tpo*, *Igf1, Il6*, *Il11*, leukemia inhibitory factor *(Lif)*, granulocyte-colony stimulating factor (G-CSF/*Csf3*), granulocyte/macrophage-colony stimulating factor (GM-CSF/*Csf2*), and transforming growth factor, beta 1 (*Tgfb1*). Importantly, the successful stromal cell lines have in common the expression of *Dlk1*, and overexpression of this factor is sufficient to enable hematopoietic support (Moore et al., 1997). Although these cell lines only maintain HSC potential and do not promote HSC expansion, some FL cells have been described to do so. Recent studies have addressed the role of specific FL populations *in vivo* or *in vitro*. It is the case of DLK1^+^ hepatoblasts that are able to expand LT-HSC around 20-fold after a 3-week co-culture period (Chou et al., 2013). This expansion seems to be contact-dependent as DLK1^+^ conditioned medium only allows expansion of ST-HSC and cells cultured in transwell inserts did not show the same expansion levels. Although promising, it is worth mentioning that these cultures were supplemented with KITL, TPO, and FLT3L and, therefore, it remains unclear which mechanisms underlie HSCs expansion in DLK1^+^ cell co-cultures. Moreover, DLK1 knockdown in human hepatoblasts results in decreased hematopoietic support *in vitro* (Gerlach et al., 2019). The role of hepatoblasts *in vivo* has been difficult to assess as transgenic mice with hepatoblast deficiencies die between E10.5 and E12.5 and studies of liver development do not usually investigate the hematopoietic compartment (Nishina et al., 1999). DLK1 has been used to identify hepatoblasts but it is also expressed by the majority of Nestin^+^ cells surrounding the portal vessels, a cell type that has been implicated as part of the FL niche (Tanimizu et al., 2003; Chou and Lodish, 2010; Chou et al., 2013; Khan et al., 2016). HSCs numbers are modestly reduced when Nestin^+^NG2^+^ pericytes are selectively eliminated and the remaining HSCs are less proliferative (Khan et al., 2016). So far, this is the only study that addresses the role of a specific cell type in HSC expansion and maintenance *in vivo.* Nestin^+^ cells are the major producers of *Cxcl12* at E14.5 when compared to Nestin^–^ cells, therefore, the reduction in FL HSCs could result from a defect in HSC expansion together with a displacement of cells into circulation. Moreover, Nestin^+^ cells seem to be a transient population, important for HSC localization around the portal vessels during FL hematopoiesis but are no longer present postnatally, a stage at which HSCs migrate and reside in the BM. These cells have also been described in adult BM and ablation of Nestin^+^NG2^+^ pericytes alters HSCs localization away from arterioles (Kunisaki et al., 2013). In the rat, fetal hepatic stellate cells were shown to express VCAM-1 and to secrete *Cxcl12* and hepatocyte growth factor *(Hgf)*, revealing a potential role for the hematopoietic and hepatic development (Kubota et al., 2007). Accordingly, mouse FL hepatic stellate cells (defined as p75NTR^+^) express a range of hematopoietic cytokines, *Csf1*, *Igf2*, *Tpo*, *Kitl*, *Epo*, *Igf1*, *Il11*, *Flt3l* and Oncostatin M (*Osm*, involved in hepatic maturation) and were therefore proposed as potential niche components (Tan et al., 2017).

To date, researchers have undertaken a cell type-directed approach, however, it is conceivable that different FL populations play distinct roles in the maintenance and expansion of HSCs and act through cellular networks. Only an unsupervised analysis of the FL constituents as a whole will shed light on the part each cell type takes in the support of hematopoiesis.

The vascular labyrinthine of the placenta (where embryonic circulation meets maternal circulation) has also been proposed as a niche for HSC expansion (Gekas et al., 2005; Ottersbach and Dzierzak, 2005; Robin et al., 2009). Human placenta-derived stromal cell lines with pericyte characteristics were shown to support *in vitro* maintenance of cord blood (CB) hematopoietic progenitors and hematopoietic cells were found in close contact with pericytes/perivascular cells in the placenta by immunostaining (Robin et al., 2009). Taken together with what has already been described for the FL (Khan et al., 2016), pericyte-like cells are likely to play a role on supporting hematopoiesis.

### Can the Adult Liver Be a Hematopoietic Site?

Extramedullary hematopoiesis (EMH) is a process in which HSPCs leave their microenvironment in the BM and establish in distinct anatomical locations wherein they continue to produce mature blood cells. Although it is a physiologic process during embryonic development (YS, AGM, FL, and fetal spleen), in the adult it only occurs in pathological settings of BM failure, myelostimulation, tissue inflammation, or abnormal cytokine production (Johns and Christopher, 2012). EMH can occur sporadically in lymph nodes, spinal cord, kidneys, gastrointestinal tract, and lung (Chiu et al., 2015). It is interesting, however, that the predominant sites of EMH are shared between the embryo and the adult: the spleen and the liver. In fact, splenic or liver hematopoiesis can still be observed postnatally in many mammals but disappears before adulthood. Hematopoietic foci in the adult liver can be found within sinusoids and in close association with macrophages (Barberá-Guillem et al., 1989). BM HSPCs co-cultured with liver sinusoid endothelial cells (LSECs) were maintained for more than 6 weeks in cytokine supplemented media, demonstrating a putative niche role of endothelial cells in the adult liver (Cardier and Barberá-Guillem, 1997). An adherent layer of liver cells has been suggested to support megakaryopoiesis by the production of TPO and B lymphopoiesis by the production of *Il7* and *Flt3l* (Cardier and Dempsey, 1998; Wittig et al., 2010). Although endothelial cells have been reported to express TPO, this cytokine is mostly produced by the liver parenchyma (hepatocytes) (Nomura et al., 1997). In the adult, EPO is produced by the kidney, although hepatocytes can also support hepatic erythropoiesis in physiological or pathological conditions (Ploemacher and van Soest, 1977; Semenza et al., 1991; Eckardt et al., 1994; Weidemann and Johnson, 2009). In conclusion, under physiologic conditions, the liver harbors low numbers of HSPCs and supports extra-medullary hematopoiesis (Taniguchi et al., 1996; Watanabe et al., 1996). Thus, it is conceivable that adult liver may keep some of its embryonic niche properties.

### Is the Development of the FL and Hematopoietic Cells Symbiotic?

In mid-gestation, embryonic liver functions as a “bag” accommodating the expanding hematopoietic system before BM development. During the temporal window in which the organ is essentially a hematopoietic tissue – from E12.5 to E16.5 (∼6–18 wpc in humans) – and the ratio of non-hematopoietic cells/total liver cells is very low, the organ’s architecture is far less complex than that of its adult counterpart. In the adult, hepatocytes are the main parenchymal cell type, organized in cords interspaced by an intricate vascular and biliary system (Figure 2H). Alongside with the massive migration of hematopoietic cells to the BM at E16.5 (Christensen et al., 2004), the liver tissue starts to mature – hepatocytes and cholangiocytes differentiate from hepatoblasts and cellular adhesion increases, creating tight hepatic parenchyma with dense hepatocyte cords (Crawford et al., 2010). Transcriptomic and proteomic analysis throughout FL development identified E15.5 as the time of onset of metabolic, detoxification and immune programs (Guo et al., 2009).

Hepatoblasts differentiate into hepatocytes starting at ∼E13.5 in mice and around 14 wpc in humans (Haruna et al., 1996; Yang et al., 2017). Single-cell transcriptomic studies along development (E10.5–E17.5) of hepatoblasts/hepatocytes/cholangiocytes (sorted based on the expression of DLK-1 and EpCAM) suggest that hepatoblast-to-hepatocyte lineage specification is the default process. Cholangiocyte specification occurs as early as E11.5 and is completed by E14.5 (Yang et al., 2017). Hepatoblasts fate decision is modulated by a gradient of Activin/TGF-β signaling, controlled by Onecut (OC) TFs (OC-1/HNF-6 and OC-2) (Clotman, 2005). Cholangiocyte-primed hepatoblasts appear in low numbers at E13.5 around the portal vein and portal sinus, forming a single-layered ductal plate at E15.5, that evolves to a double-layer by E16.5 (Figures 2D,E), characterized by CK19 and CK8 cytokeratins and β2 integrin (CD18) (Van Eyken et al., 1988; Tanimizu et al., 2009). At this stage, CK19 expression becomes specific to ductal plate cells (Van Eyken et al., 1988). Focal dilations between the two ductal plate cell layers give rise to the bile ducts (Figures 2F,G), whereas the remaining tissue progressively regresses (Clotman et al., 2002). This ductal plate remodeling involves tubulogenesis (Antoniou et al., 2009) and apoptosis (Terada and Nakanuma, 1995). Around birth, the portal mesenchyme encircles the cells of the ductal plate (Swartley et al., 2016). Hepatoblasts located away from the portal vein will gradually differentiate into hepatocytes and by E17 start to exhibit a characteristic polarized epithelial morphology disposed in hepatic cords alongside the bile canaliculi (Zorn, 2008). Because cholangiocyte differentiation occurs along the hilum-to-periphery axis, different maturation states can be observed at a given developmental time (Yang et al., 2017). Three-dimensional reconstructions of serial cross-sections/whole-mount immunostained FL and carbon ink injection have been used to disclose the morphogenesis of intrahepatic bile ducts (Vestentoft et al., 2011; Takashima et al., 2015; Tanimizu et al., 2016).

Kinoshita et al. (1999) showed that hematopoietic cells expand when cultured in a monolayer of primary fetal hepatic cells (in presence of hematopoietic cytokines) and that the addition of OSM suppresses *in vitro* hematopoiesis, by inducing the maturation of the hepatic cells. Since hematopoietic cells produce OSM, its expansion increases the local OSM concentration, consequently promoting hepatic development (Kamiya et al., 1999). It was hypothesized that a metabolically active liver no longer supports hematopoiesis (Miyajima et al., 2000). However, it is still not clear whether the displacement of the hematopoietic cells out of the FL facilitates liver maturation or if the changes in the microenvironment no longer support hematopoiesis.

Impaired hematopoiesis in *c-Myb* mutant (Mucenski et al., 1991) or *Ubc*^–/–^ mice (Ryu et al., 2012) and abnormal erythropoiesis in *Rb*-deficient mice (Lee et al., 1992) also results in impaired liver growth. However, the early embryonic lethality (at around E15) has hindered the analysis of the impact of the hematopoietic compartment in liver development.

The vasculature remodeling at the end of gestation was also correlated with the rapid loss of HSCs in the postnatal liver (Khan et al., 2016). After birth, the portal vein no longer receives blood from the vitelline vein, collecting the blood from the gut, draining it to the central vein and the hepatic artery arises. With the ligation of the umbilical inlet, the portal vessels acquire a vein phenotype and lose the periportal pericytes (Khan et al., 2016).

## Hematopoietic Exit From Fl and Establishment in the BM

### What Makes the Hematopoietic System Move? How Is the BM Niche Established?

The BM is the ultimate destination of the hematopoietic system journey in the embryo, being the production hub for blood cells throughout life. LSK cell homing and colonization of mouse BM was reported at E15.5–E16.5, coinciding with the fetal bone marrow (FBM) vascularization (Gekas et al., 2005; Coşkun et al., 2014; Cao et al., 2019), although LT-HSC activity cannot be detected before E17.5 (Christensen et al., 2004). In mice, HSCs migrating out of the FL also seed the spleen, starting at E15.5, with HSC activity still being detected a few weeks after birth (Wolber et al., 2002; Christensen et al., 2004; Bertrand et al., 2006). In humans, although early colonization of fetal long bones was reported at ∼10 wpc (Charbord et al., 1996), the hematopoietic shift from the FL to the FBM occurs later, around 20 wpc (Figure 1).

Coşkun et al. (2014) showed that hematopoietic progenitor cells reside in the vascularized regions of fetal long bones, stage at which they are still proliferative. Previous work showed that in the BM, HSCs are cycling during the first 3 weeks after birth and become quiescent thereafter (Bowie et al., 2006). The shift to a quiescence state seems to be dependent on the cellular composition of the microenvironment (Coşkun et al., 2014). LSK cells isolated from *Osx*^–/–^ FBM [that lack osteolineage cells (Nakashima et al., 2002) and some stromal populations (Mizoguchi et al., 2014)] form multi-lineage colonies *in vitro*, but fail to repopulate transplanted recipients (Mizoguchi et al., 2014). LSK cells exhibited dysregulated cell cycle progression and defective homing ability, suggesting that osteolineage and/or mesenchymal cells are necessary to establish and sustain BM LT-HSCs (Coşkun et al., 2014). Additionally, E15.5 CD105^+^Thy1^–^ mesenchymal progenitors transplanted under the kidney capsule give rise to donor-derived chondrocytes and can create an ectopic niche with the recruitment of host-derived marrow and HSCs, evidencing the importance of endochondral ossification for HSC niche formation (Chan et al., 2009).

Osteopontin (OPN), also known as secreted phosphoprotein 1 (SPP1) has been suggested to be an important niche factor in BM. Its concentration in stromal cells is inversely correlated with HSC proliferation in the adult BM (Stier et al., 2005). The OPN dominant form thrombin-cleaved osteopontin (trOPN) is highly expressed in FBM (at the trabecular bone surface), but neglectable-to-none in the early-mid gestation FL (Cao et al., 2019). trOPN receptor α4β1 integrin is upregulated in fetal compared to adult HSCs. The differential concentration of the divalent metal cations, Ca^2+^, Mg^2+^, and Mn^2+^ between FL and FBM, being highly prevalent in the latter, was assumed to activate α4β1 in HSCs, possibly hindering their expansion in the BM (Cao et al., 2019).

CXCL12 expression in the vicinity of vascular endothelial cells in FBM supports the hematopoietic colonization of the organ (Nagasawa et al., 1996; Ara et al., 2003). HSCs isolated from CXCL12^–/–^ embryos failed to colonize the BM in long-term repopulation assays whereas their migration ability could be rescued by enforced expression of CXCL12 under the control of vascular-specific Tie-2 regulatory sequences (Ara et al., 2003). Moreover, the CXCL12-mediated migration of HSPCs *in vitro* seems to be enhanced by the presence of KITL, indicating an additive effect, only found in fetal HSCs (Christensen et al., 2004). This suggests an important role of KITL for HSC seeding and homing during embryonic development.

*CXCL12-GFP* knock-in mice allowed the identification of CXCL12-abundant reticular (CAR) cells, a population with high expression of CXCL12, distributed near SECs and at a lower extent near the endosteum (Sugiyama et al., 2006). These cells show *in vitro* potential to differentiate into adipocytic and osteoblastic lineages (Omatsu et al., 2010). Specific markers have been identified for mesenchymal cells in BM. Leptin receptor (Lepr) is highly enriched in *Scf-GFP*-expressing perivascular stromal cells (Ding et al., 2012) and Lepr^+^ cells were shown to largely overlap with CAR cells (Zhou et al., 2014). A rare population of Nestin^+^ cells that contains all of the fibroblastic CFU (CFU-F) activity within the mouse BM, capable of generating mesenspheres (mesenchymal spheres) *in vitro*, multipotent and with self-renewal potential was identified as a mesenchymal stem cell (MSC) population (Méndez-Ferrer et al., 2010). Later, two different Nestin-GFP populations were discriminated based on the fluorescence intensity by microscopy. Rare quiescent *Nestin*-*GFP*^*bright*^ cells, positive for the pericyte marker NG2 and α-smooth muscle actin, are enriched for CFU-F activity and express *Cxcl12* and *Kitl*. These cells are located alongside arterioles, close to dormant HSCs (Kunisaki et al., 2013), and associated with sympathetic nerves, that regulate their CXCL12 expression through the β_3_-adrenergic receptor (Méndez-Ferrer et al., 2010). *Nestin*-*GFP*^*dim*^ cells have a reticular shape, are mitotically active and line sinusoids, largely overlapping with Lepr^+^ cells (∼80%) (Kunisaki et al., 2013). Selective ablation of mouse Nestin^+^ cells (Méndez-Ferrer et al., 2010) or CAR cells (Omatsu et al., 2010) significantly impacts the maintenance of HSCs. The structural differences of the blood vessels and perivascular populations seem to be associated with heterogeneity in HSC function. Besides, the selective deletion of *Cxcl12* from arteriolar NG2^+^ cells, but not sinusoidal Lepr^+^ cells, significantly reduced the HSCs compartment in the BM and a similar effect was observed by deletion of *Kitl* in LepR^+^; but not NG2^+^ cells, evidencing the differential contribution of perivascular populations in the cytokine production (Asada et al., 2017). BM microenvironment is illustrated in Figure 4.

Nestin^+^ cells can be prospectively isolated using PDGFRα and CD51 markers in the mouse and human fetal and adult BM (Pinho et al., 2013) and show MSC’s properties and enrichment of *Cxcl12*, *Vcam1*, *Angpt1*, *Opn*, and *Scf* genes. Of note, in humans, these cells represent a small subset of CD146^+^ cells (Pinho et al., 2013), the latter harboring all the CFU-F activity in BM (Sacchetti et al., 2007). Co-culture of human PDGFRα^+^CD51^+^ mesenspheres with human FBM CD34^+^ cells in a serum-free, but cytokine supplemented (TPO, SCF, FLT3L) culture media can expand MPPs that engraft immunodeficient mice (Pinho et al., 2013).

In the FL, Nestin-GFP^+^NG2^+^ cells, associated with portal vessels, form a niche promoting HSC expansion during the FL development that is no longer found after birth. Concomitantly, the phenotype of the portal vessel transits from Neuropilin-1^+^Ephrin-B2^+^ arterial to EphB4^+^ venular vessels (Khan et al., 2016). This role of Nestin^+^NG2^+^ cells in FL is opposite to that in BM, where Nestin^+^NG2^+^arteriolar pericytes were proposed to maintain HSC quiescent (Kunisaki et al., 2013).

## Unresolved Questions – *Ex vivo* Expansion of HSCs

Hematopoietic stem cells are the only cells of the hematopoietic compartment with the potential to replenish all mature blood cells and to divide without triggering differentiation programs, a process known as self-renewal. The mechanisms conveying these properties have been under investigation over the last 60 years, however, to date, they remain poorly understood. The concept that specific BM niche/microenvironment components regulate the fate of HSCs has been proposed by many authors. Cordeiro Gomes et al. (2016) analyzed not only HSCs but also different hematopoietic progenitors and found both HSCs and MPPs locate near or in contact with the same mesenchymal progenitor CAR-cells, expressing CXCL12 and SCF, fundamental to maintain the HSC pool and IL-7 that acts as a short-range signal for lymphoid differentiation. These observations suggest that both maintenance and multilineage differentiation are locally regulated by the same niche (Cordeiro Gomes et al., 2016). In the FL, HSCs expand considerably and differentiate, producing different mature lineages. If a given stromal population is also involved in the regulation of both processes, by the expression of various cytokines, or different cell populations contribute with distinct cytokines is still unresolved.

Hematopoietic stem cell transplantation is a widely used cell therapy intervention in the treatment of hematologic, autoimmune and genetic disorders. However, this therapy is still associated with high mortality rates, mainly due to infection, graft-versus-host disease (GvHD) and organ dysfunction, urging the need for improvement (Tanaka et al., 2016). The most common source of HSCs for transplantation is the BM or mobilized circulating HSPCs. However, matching of major histocompatibility complex antigens is needed to avoid GvHD (Schuster et al., 2012; Walasek et al., 2012). CB can be successfully used as a source of partially mismatched HSCs, as it is readily available through CB banks and elicits low levels of GvHD. Low numbers of HSCs in CB and consequent absent or delayed reconstitution leads to post-transplantation infections, limiting the use of CB in adult patients (Ruggeri et al., 2014).

*In vitro* generation of HSCs would overcome some of the current clinical difficulties that transplantation faces, however, despite countless efforts to derive HSCs from pluripotent stem cell sources, generation of HSC *in vitro* has not yet been achieved – reviewed in Freire and Butler (2020). Another possibility to obtain higher numbers of HSCs would be to expand them *ex vivo* prior to transplantation. Distinct cytokine/growth factor cocktails have shown promising for expansion of HSCs, yet, limited success was reported in clinical studies due to a lack of LT-HSC expansion and rather proliferation of downstream progenitors together with undesirable stem cell differentiation – reviewed in Kumar and Geiger (2017) and Tajer et al. (2019). Cytokines currently used for *ex vivo* HSC expansion include KITL, TPO, IL-3, and FLT3L. *Kitl* and *Tpo* knockout mice show normal fetal development but reduced HSC numbers in the adult, suggesting that these cytokines are important for stem cell survival and proliferation in adulthood but might not be the drivers of HSC expansion during the embryonic period (Fujita et al., 1989; de Sauvage et al., 1996). There is growing evidence that the physical and mechanical properties of the microenvironment could impact on HSC decisions – reviewed in Kumar and Geiger (2017). A combination of cytokines with stroma derived ECM components – fibronectin and collagen – has shown encouraging results (Wohrer et al., 2014; Wilkinson et al., 2019). Moreover, culture of BM HSPCs in tropoelastin, the most elastic biomaterial known, induces a sixfold increase of LSK cells without supplementation with cytokines, suggesting that tropoelastin mediates a similar effect in survival and proliferation of LSK cells and its use could replace exogenous cytokines (Holst et al., 2010). Most cytokines studied have a role in HSC function in the BM, a site where HSC expansion does not occur in physiological conditions. The same cytokines have been found in FL stroma, but whether these are responsible for fetal HSC expansion is not known. Most likely, the FL expansion of newly generated HSCs results from intrinsic cellular properties together with a suitable microenvironment, physical cues included. Efforts have been made to replicate the embryonic microenvironment where HSCs expand, particularly by co-culture with FL hepatic or mesenchymal cells (Wineman et al., 1996; Chou and Lodish, 2010; Chou et al., 2013; Khan et al., 2016). Most strategies used so far focused on specific cell populations, either hepatoblasts or mesenchymal cells, but overlooked the possibility that distinct populations might need to interact. Only the co-culture of HSCs in a system where all the cytokine-expressing populations of the FL are present would reproduce the FL microenvironment. Such a system where hepatoblasts, endothelial and mesenchymal cells are cultured together in a 3D aggregate has already been devised, but no co-culture with hematopoietic cells has been attempted (Takebe et al., 2015). Liver cell culture models have been extensively developed for pharmacological and toxicological research or as a source for transplantation, to obtain an *in vitro* system that resembles a mature liver – reviewed in Godoy et al. (2013), and for that reason are not suitable for recreating the FL niche environment.

Whereas the FL interactions between different cell types have been correlated with their contribution to the massive transient expansion of the hematopoietic system, other niche factors need to be addressed. Sigurdsson et al. (2016) reported the role of FL bile acids (BAs) as chemical chaperones, critical to sustain high protein production by expanding LT-HSCs without triggering endoplasmic reticulum (ER) stress. Inhibiting the biosynthesis of BAs *in vivo* resulted in reduced numbers of Lin^–^, LSKs, and LSK CD48^–^CD150^+^ in the FL, with no apparent effect in the number of HSCs in the mother’s BM (Sigurdsson et al., 2016). Comparison of FL and BM HSCs transcriptomes demonstrated that FL HSCs metabolism relies on oxygen-dependent pathways, which may be a requirement for extensive energy production during expansion. Contrary to BM HSCs, FL HSCs use oxidative phosphorylation (aside from glycolysis), have higher number of mitochondria and up regulate genes associated with antioxidant and DNA repair pathways, that are speculated to confer protection from reactive oxygen species-mediated (geno)toxicity (Manesia et al., 2015).

Exploring the complexity of HSC niches (cellular composition, cytokine and growth factors milieu, physical properties, oxygen availability, etc.) will improve into our understanding of HSC self-renewal capacity (recently reviewed by Wilkinson et al., 2020), that is currently insufficient to devise efficient strategies for HSC expansion *ex vivo*.

## Concluding Remarks

Distinct studies have identified and characterized FL stromal populations that may contribute with specific cues, enabling the HSC expansion in this organ. Despite the efforts, the role of the FL microenvironment has not been directly shown. The possibility that HSCs proliferate exclusively due to intrinsic properties is however remote. Therefore, a model in which distinct populations cooperate is conceivable. An analysis that contemplates such complex multicellular networks has not been attempted. This review aimed to compile information on the cellular populations that could signal to HSCs during development, highlighting the advances and the unresolved questions in the field.

## Author Contributions

FS-d-S and MP: conceptualization and writing. AC: discussion and revision. PP-d-Ó: conceptualization and revision. All authors contributed to the article and approved the submitted version.

## Conflict of Interest

The authors declare that the research was conducted in the absence of any commercial or financial relationships that could be construed as a potential conflict of interest.
